# Online Assessment for Pathology Residents during the COVID-19 Pandemic: Report of an Experience

**DOI:** 10.30699/ijp.2020.129558.2425

**Published:** 2020-10-26

**Authors:** Alireza Abdollahi, Ali Labbaf, Mahboobeh Khabaz Mafinejad, Maryam Sotoudeh-Anvari, Farid Azmoudeh-Ardalan

**Affiliations:** 1 *Department of Pathology, Imam Hospital Complex, School of Medicine, Tehran University of Medical Sciences, Tehran, Iran.*; 2 *Department of Emergency Medicine, Imam Khomeini Hospital Complex, Tehran University of Medical Sciences, Tehran, Iran.*; 3 *Education Development Center, Health Professions Education Research Center, Tehran University of Medical Sciences, Tehran, Iran.*; 4 *Department of Pathology, Children's Medical Center, School of Medicine, Tehran University of Medical Sciences, Tehran, Iran*

**Keywords:** Coronavirus, COVID-19, Electronic exams, Pathology

## Abstract

**Background & Objective:**

The world is facing COVID-19 pandemic, and medical education system and consequently the evaluation of students at different levels have been overshadowed. Residency students are among those affected. In the present study, we aim to share our experiences regarding holding exams for pathology residents before and during the pandemic.

**Methods:**

This cross-sectional study was carried out in Tehran University of Medical Sciences. The online exam, which consisted of 30 multiple-choice questions, was designed and held in April 2020 to evaluate pathology residents. To assess the quality of the exam, indices such as the number of questions, highest and lowest scores, the average score, the standard deviation, the variance, Cronbach’s alpha reliability coefficient, standard error of measurement, discrimination index, difficulty index, number/percentage of questions on three difficulty levels of easy, normal, hard were reviewed and analyzed.

**Results and Discussion:**

The average score of the participants in the January exam which was held in the university’s exam center was 16.23 (±5.03), while the average score in the online exam which was held after the onset of the pandemic was 20.86 (±5.18). The average discrimination indices in the first and second exams were 0.36 and 0.38, respectively, and the average difficulty indices in the first and second exams were 0.54 and 0.70, respectively. We found the administration of this online examination would be a positive experience. By sharing it, we hope to pave the way for similar ventures in the other departments.

## Introduction

We are now facing an all-out pandemic of COVID-19 (1). The disease’s quick spread and its turning into a pandemic, in addition to serious consequences on areas of healthcare, economy, and society (2-4), have posed new challenges for academic institutes all over the world (5, 6). One of the main concerns of instructors and students is the way this pandemic might affect the quality of medical training programs (7-9). A review of the recent studies on the topic reveals that different medical science institutes have been obligated to modify their training methods in response to the new circumstances (10). Consequently, many of the pre-defined aspects of training programs, such as in-person training sessions and proctored exams have undergone changes. Following the COVID-19 pandemic, there has been a growing inclination towards teaching theoretical subjects through synchronized and a-synchronized methods (9), having interactive sessions in the form of small educational groups on online platforms (11), and holding virtual clinical and theoretical exams (12, 13).

Training medical residents has been affected by this pandemic as well, and given the new circumstances, these students have seen their received knowledge, their attitudes towards learning, and their performances undergoing unforeseen changes (14). Training residency students is more important compared to other learners, because besides taking part in their training activities, these students are expected to lend a hand in taking care of COVID-19 patients (15).

In our new reality of “The World with Viruses,” it seems like we have little choice but to use the technological advances we have on hand, such as various virtual training platforms, and to use this opportunity to gain useful experience in such new frontiers as online examinations (16). There is little doubt that such new ventures will be faced with difficulties and uncertainties. Meanwhile, some examinations, including high-stake ones, have been simply cancelled or delayed due to lack of necessary infrastructures. A point of note is that such cancellations and delays might turn out to be long-turn necessities. As such, we need to come up with a plan to manage such challenges (17).

The Pathology Department at Tehran University of Medical Sciences was established over eight decades ago in 1934, and over 1000 of its graduates have been in service since. Members of the faculty have always been on a mission to provide an up-to-date educational program for their residents and students, and have achieved considerable success on their path. The electronic examination center of Tehran University of Medical Sciences was stablished at Children’s Medical Centre around 2018; therefore, given the circumstances following the Pandemic, the faculty members at the Pathology Department decided to hold online monthly exams, giving the students at the residency program a chance to prepare for their academic certificate and credentials examinations which were also planned to be held electronically in the future. By so doing, using high-quality histopathological illustrations became possible and paper consumption was reduced. In the present study, our aim is to share our experience of holding clinical and surgical pathology examinations using online platforms during the COVID-19 pandemic.

##  Materials and Methods

This cross-sectional study was carried out in Tehran University of Medical Sciences in 2020. Having the COVID-19 pandemic in mind, and following several meetings, faculty members of the pathology department decided to use online exams to evaluate the performance of their students in making diagnoses based on pathological images. Accordingly, based on a pre-defined schedule for exams, the necessary planning was carried out to hold an online exam during the COVID-19 pandemic. To familiarize the faculty members with the exam procedure on online platforms, basic training was carried out. Questions were designed by two faculty members who were chosen based on their area of expertise for each examination, and then, based on the assigned references, 30 multiple-choice questions with their associated illustrations were prepared for the exam. The total allocated time for an exam was 30 minutes.

All the people involved were consulted and a decision was reached to hold the exam online. The residents took their exams at the hospitals where they were working or at their homes. One day before the date of the exam, the students were asked to try signing in to the platform using a provided link to a mock exam. Encountered errors were then resolved by the technical support team where needed. Furthermore, the students were informed about how they should access the exam platform on time, how the questions would be presented and randomized, the duration of the exams, and the scoring rules. The following indices were reviewed and analyzed to determine the quality of the exam: the number of questions, the highest and the lowest scores, the average score, standard deviation, variance, Cronbach’s alpha reliability index, standard error of measurement, discrimination index, difficultly index, number/percentage of questions on three difficulty levels of easy, normal, hard. After the exam, the residents’ scores and an analysis of the questions were delivered to the person responsible for holding the exam, and once a review of the analysis had been conducted, the results were delivered to the residents and their instructors.

##  Results and Discussion

All the aspects of the exam were similar to those of the previous one, except for the method of administration. Accordingly, it was decided to compare the results of the April exam to the results of a similar month. Given the fact that the residents complete their histological studies in their first year before beginning to take their pathological exams in January, the results of the January exam were compared to the results of the April exam. The outcome of the analysis of the quality indices of these exams has been summarized in Table 1.

**Table 1:** A comparative analysis of quality indices of residents’ examination before and after the COVID-19 pandemic

	January	April
Number of participants	74	75
Number of questions	30	30
Highest score	30	30
Average score	16.23	20.86
Standard deviation	5.03	5.18
Variance	25.36	26.86
KR20 reliability	0.75	0.80
Cronbach’s alpha	0.75	0.80
Standard error of measurement	0.92	0.94
The mean coefficient of determination	0.36	0.38
The mean coefficient of difficulty	0.54	0.70
Number (percentage) of easy questions	6 (20%)	18 (60%)
Number (percentage) of normal questions	21 (70%)	10 (33%)
Number (percentage) of hard questions	3 (10%)	2 (7%)

As demonstrated, the Cronbach’s alpha to assess the reliability of these exams is in the 0.7-0.9 range (18), meaning both exams are in the desired range of reliability. We know that, in a norm-referenced assessment, the closer the difficulty index to 0.5 the better (19). However, the online exam was administered as a criterion-referenced assessment to determine whether these students had achieved a minimum of competency with reference to a set standard, and as such, in this particular case, a number closer to 1 is preferable (20). Although the difficulty index went up and was closer to 1 in April, which means that the exam has been on the easier side in April, we should keep the security of the April exam at bay, as the security measures were not as tight as the January due to the different nature of these exams. Additionally, the percentages of easy, normal and hard questions were different between the two exams which can further eschew the results. Based on these findings, we can argue that designing the questions should be based on higher levels of Bloom’s taxonomy. The determination index which measures the suitability of an examination for differentiating strong students from weak ones is considered acceptable if the value is above 0.3 (21), as it was in both of the exams under consideration in the present study. Higher values of determination index are not to be expected in a criterion-referenced assessment, and the findings in the present study reflect this fact (22).

The shortcomings of this study include the fact that, despite the aforementioned measures, the security of the April exam could not be guaranteed; a number of participants faced internet connection difficulties while taking the April exam and were worried about the time limit; and the sample of this study consisted of a small number of students in a particular field. We recommend taking these limitations and the circumstances of the exam into account when interpreting the findings of this study. In future studies, further security measures should be utilized to ensure the soundness of examinations.

We found the administration of this online exam a positive experience. By sharing it, we hope to make similar ventures in other departments easier. All the pathology residents managed to take part in the online exam, even those who could not be present in the university due to travel restrictions during the pandemic. This online exam was held using a user-friendly platform that did not put extra pressure on the participating students, many of which found the educational aspect of the exam useful. The excellent technical support during the examination ensured the smoothness of the experience for these students. Minimum traffic was guaranteed by holding this online examination, providing a much-appreciated safety for our students during the pandemic. As a further step, we suggest a questionnaire be designed to ask the opinion of the residency students.

## Conclusion

Once the necessary executive precautions are observed to ensure an online exam’s security, it can be used to assess the knowledge and inferring skills of the pathology residents during the COVID-19 pandemic. Additionally, if Bloom’s taxonomy principles are adopted to design the questions, and if an apt post-examination analysis is carried out, online exams may be improved to a point where they can be reliably used when residents cannot be physically present to take their exams.
